# Prevalence and correlates of soil-transmitted helminths in schoolchildren aged 5 to 18 years in low- and middle-income countries: a systematic review and meta-analysis

**DOI:** 10.3389/fpubh.2024.1283054

**Published:** 2024-03-21

**Authors:** Ritik Agrawal, Sweta Pattnaik, Jaya Singh Kshatri, Srikanta Kanungo, Nityananda Mandal, Subrata Kumar Palo, Sanghamitra Pati

**Affiliations:** ICMR-Regional Medical Research Centre, Bhubaneswar, Odisha, India

**Keywords:** soil-transmitted helminths, NTDs, school-going children, LMICs, meta-analysis

## Abstract

**Background:**

According to the Global Burden of Disease (GBD) 2019 report, up to 1.5 million disability-adjusted life years (DALYs) are lost due to soil-transmitted helminths (STHs), and 5.9 million people are at risk of acquiring STHs. Regions with the highest prevalence of STH infections include Sub-Saharan Africa, China, South America, and Asia. While there are numerous fragmented studies on STH, comprehensive information on the prevalence and geographic distribution of different species, as well as their regional variations in the context of STHs is limited. The present systematic review and meta-analysis study attempts to provide a summary of the prevalence, geographical variation, and determinants of STHs among schoolchildren aged 5 to 18 years.

**Methods:**

An extensive literature search was carried out using PubMed, Embase, Cinhal, and Psychinfo for studies published between 1999 and 2022 that reported the rate of STH infection in school-going children aged 5–18 years. A random effects model was employed in this meta-analysis due to expected heterogeneity. Subgroup analysis was carried out based on sex and STH species because of expected geographical variation.

**Results:**

A total of 19,725 of the 49,630 children examined were infected with STH, yielding an overall pooled prevalence of 37.16% (95% CI: 29.74–44.89). The prevalence was highest in the Western Pacific region at 50.41% (95% CI: 33.74–67.04) followed by Europe at 39.74% (95% CI: 20.40–61.0) and Africa at 37.10% (95% CI: 26.84–47.95). *Ascaris lumbricoides* was found to be the most prevalent helminth with a prevalence of 24.07% (95% CI: 17.07–31.83).

**Conclusion:**

The Western Pacific region is classified as a High-risk Zone (HRZ), while Southeast Asia, Africa, Europe, and the Eastern Mediterranean are classified as moderate-risk zones (MRZs). We found a 12% reduction in the pooled prevalence of STH infection from 1999 to 2012. *Ascaris lumbricoides* was the predominant species among schoolchildren. Mass Drug Administration (MDA) of Albendazole tablets and improved water, sanitation, and hygiene (WASH) practices are effective in controlling and preventing STH. Ensuring their implementation and access is crucial to addressing the problem.

**Systematic review registration:**

https://www.crd.york.ac.uk/prospero/#loginpage, CRD42022333341.

## Background

Soil-transmitted helminths (STHs) are a group of neglected tropical diseases (NTDs) that affect over 1.5 billion people (24% of the world’s population) as per the World Health Organization (WHO) (1). According to the Global Burden of Disease (GBD) 2019, the reported global Disability-Adjusted Life Years (DALYs) due to STHs were as high as 1.5 million (2, 3). Additionally, approximately 5.9 billion people worldwide are at risk of acquiring STHs (4). Of the various age groups, children are the most vulnerable and at the highest risk of acquiring STH infections because of their immature immune system, tendency to play on the ground, and poor hygiene practices (5, 6). Furthermore, they are more susceptible to the pathological consequences of STH infections. More than 260 million preschool children, 654 million school-age children, and 108 million adolescent girls are estimated to live in regions where transmission of STH parasites is intense (1).

STHs are a group of parasites including *Ascaris lumbricoides*, *Trichuris trichiura, Hookworms (Ancylostoma duodenale and Necator americanus)*. Infections caused by STHs have a greater impact on poor and disadvantaged communities that lack access to clean water, sanitation, and hygiene facilities in tropical and subtropical regions (7). The regions with the highest prevalence of STH infections are Sub-Saharan Africa, China, South America, and Asia (8). This is due to the fact that the life cycle of STHs requires fecal contamination of the soil, and as a result, the burden of their infection is higher in areas where hygiene practices are poor and sanitation facilities are scarce.

STHs are primarily transmitted through the feces of infected individuals, which contain eggs or larvae. Adult worms living in the gut of an infected person can produce large quantities of eggs daily, leading to contamination of the environment and food sources that lack proper sanitation (9). The tropical and sub-tropical climates in certain regions are suitable for the survival of STH eggs and larvae, as they require warm temperatures and moist soil (10, 11). In children, STH infections have profound implications, affecting their nutritional well-being, physical growth, and overall health. These infections bring about a range of issues such as gut blood loss, impaired nutrient absorption, diminished appetite, and anemia, all of which contribute to hindered cognitive development (4). Furthermore, the impact of STH infections on cognitive performance and learning abilities cannot be underestimated. Various studies have shed light on the adverse effects of these infections on children’s health, manifesting as stunted growth, anemia, malnutrition, and decreased school attendance (12, 13) and involve a loss of disability-adjusted life-years in affected children (4, 14, 15).

In 2012, the World Health Organization (WHO) released a detailed plan to address Neglected Tropical Diseases (NTDs) with the goal of eliminating them by 2020. One of the key strategies developed was the Mass Drug Administration (MDA) approach, which aimed to provide medication to at least 75% of affected individuals where STHs were present (16). The WHO subsequently set a target of STH elimination by 2030 (17). To this end, it is important to have evidence of the burden of STH, especially in low- and middle-income countries (LMICs), due to its high prevalence.

Nevertheless, numerous fragmented studies have been carried out to assess the prevalence of STHs among preschool children (PSAC) and SAC in different countries, but comprehensive data on the prevalence, geographic distribution of different species, patterns as well as regional variations among different regions of the country are lacking in the context of STHs. As a result, the current study aimed to provide a summary of the prevalence, geographical variation, species-specific prevalence, and various determinants of STHs among schoolchildren aged 5 to 18 years, which could assist the government and other relevant bodies in prioritizing and developing context-appropriate strategies to control and prevent STH infection among school-age children.

## Methods

### Protocols and standards

This systematic review was prospectively registered with the International Prospective Register of Systematic Reviews (Registration ID: CRD42022333341) which was registered on 13th June 2022 (18). It was conducted and reported according to the Preferred Reporting Items for Systematic Reviews and Meta-analysis (PRISMA) guidelines (19).

### Eligibility criteria

Original observational studies reporting prevalence and determinants of STH, studies with a population including schoolchildren aged 5 to 18 years, conducted either in a community-based setting or primary studies from LMICs between 1999 and 2022 were included. The diagnostic methods used for STH detection (such as fecal concentration, Kato-Katz method, McMaster, and the flotac technique) were evaluated. Additionally, all included studies were published in English.

Research papers that reported the prevalence of STH among (human immunodeficiency virus, TB, malaria, and other health issues), preschool children, and students undergoing treatment were higher, thus they were excluded from the analysis, as were hospital-based prevalence studies. Additionally, systematic reviews, randomized controlled trials, and reviews were also excluded from this study.

STH infections can be affected by other health issues, especially in people with weakened immune systems, such as those with HIV. These interactions can make it difficult to understand how common STH infections are in the general population. So, this is what we are focusing on to get a clearer picture of how widespread STH infections are by themselves, without these other factors coming into play.

We also excluded preschoolers and students under treatment, focusing on untreated individuals in the general population. Hospital-based prevalence studies were excluded due to their potential bias toward severe cases. Additionally, systematic reviews, randomized controlled trials, and reviews were excluded to ensure a clear focus on primary cross-sectional studies. These decisions were made to provide a more accurate representation of the prevalence of STH infections in the general population and to streamline the analysis for the research objectives.

### Information sources and search strategy

We searched the medical literature databases to make our search exclusive. A comprehensive search was carried out using the electronic databases Medline through PubMed and Embase along with Cinhal and Psychinfo. The basic search syntax comprised four main concepts: prevalence, soil-transmitted helminths, school-going children, and low- and middle-income countries. The search was conducted in Medline and PubMed using search terms such as “STH,” “Soil-Transmitted Helminth,” and “Animal helminthiases” along with medical subject heading (MeSH) terms identifying soil-transmitted Helminthes such as “helminth,” “Ascaris lumricoide,” “trichurias,” “*Necator americanus*,” “*hookworm*,” “*hookworm* infection.” “*Trichuris trichiura*,” “roundworm” and “ancyclostoma duodenale” were searched along with LMICs using the individual names of the country and “school-going children.” These relevant keywords were combined by using the Boolean operators “AND” and “OR.” However, for other databases the terms were used together with other keywords to make the search strategy more comprehensive. The search strategy was then refined to include only data from LMICs. The detailed search strategy is provided in Supplementary material S1.

### Study selection, data extraction, and synthesis

The researchers combined and filtered studies from multiple databases to remove duplicates. Three reviewers (RA, JSS and SP) independently screened the articles based on their titles and abstracts, categorizing them as relevant, irrelevant, or uncertain. If all three reviewers marked an article as irrelevant, it was eliminated. The full texts of the included articles from the primary screening were then reviewed by the same three independent reviewers to determine eligibility based on the inclusion and exclusion criteria. Disagreements were resolved by consensus with the help of other reviewers (JSS, SPati, and SPalo).

Relevant data from the studies were collected using a pre-designed data extraction sheet and entered by two independent reviewers (RA and SP). Two reviewers (JSS, SPati and SPalo) checked the data for any discrepancies, with inconsistencies resolved by consensus of the entire team. If any data were unclear, the team contacted the respective authors for clarification. After determining the relevance of the articles, the full texts were downloaded for detailed review. We extracted information on the name of the first author and year of publication, study design, sex, region of study, country, laboratory method for parasite identification, total sample size, species-specific prevalence, the number of positive cases for STHs, and a quality assessment for each research paper included in this study. We have adapted the Johanna Bricks Institute (JBI) checklist to evaluate the quality of each study. Scoring was achieved by assessing specific questions related to the study, and each entity was scored accordingly. It is crucial to remember that the JBI checklist doesn’t have a comprehensive numerical scoring system and set score criteria. Rather, the emphasis is on assessing the study’s quality by determining if it satisfies the checklist’s requirements (20). The detailed grading and method adopted for quality assessment of the included research studies is presented in Supplementary material S2.

### Statistical analysis

The findings were summarized as a quantitative summary of the evidence, with pooled prevalence estimates representing the quantitative summary. We used R Software version 4.2.2 for data analysis. Forest plots were used to estimate the pooled effect size and the effect of each study, along with their confidence intervals, to provide a visual summary of the data. A random effects model was employed in this meta-analysis due to expected heterogeneity. The *I*^2^ statistic was used to assess the variability between studies; this statistic can take values between 0 and 100%, with high values indicative of strong heterogeneity. We anticipated high heterogeneity due to the nature of the selected studies such as nationally representative samples vs. primary studies with relatively smaller sample sizes; and age group considered in the study. Given the anticipated regional diversity and the likelihood that socioeconomic circumstances may differ considerably between studies, subgroup analysis was conducted depending on STH type and gender. The asymmetry of the funnel plot was used to detect publication bias; furthermore, quantitative assessments of the potential for publication bias were conducted using Egger’s regression and Begg’s correlation tests. Visual inspection for publication bias by plot symmetry with a *p* value of <0.05 was considered statistically significant (Supplementary material S3). The results of Begg’s correlation test and Egger’s regression test were found to be non-significant (*p* value of ˃0.05).

### Patient and public involvement

We did not involve any patients or the public during the designing, conducting, reporting, or dissemination plans of our research.

## Results

### Literature searches and selection

**Figure 1:**
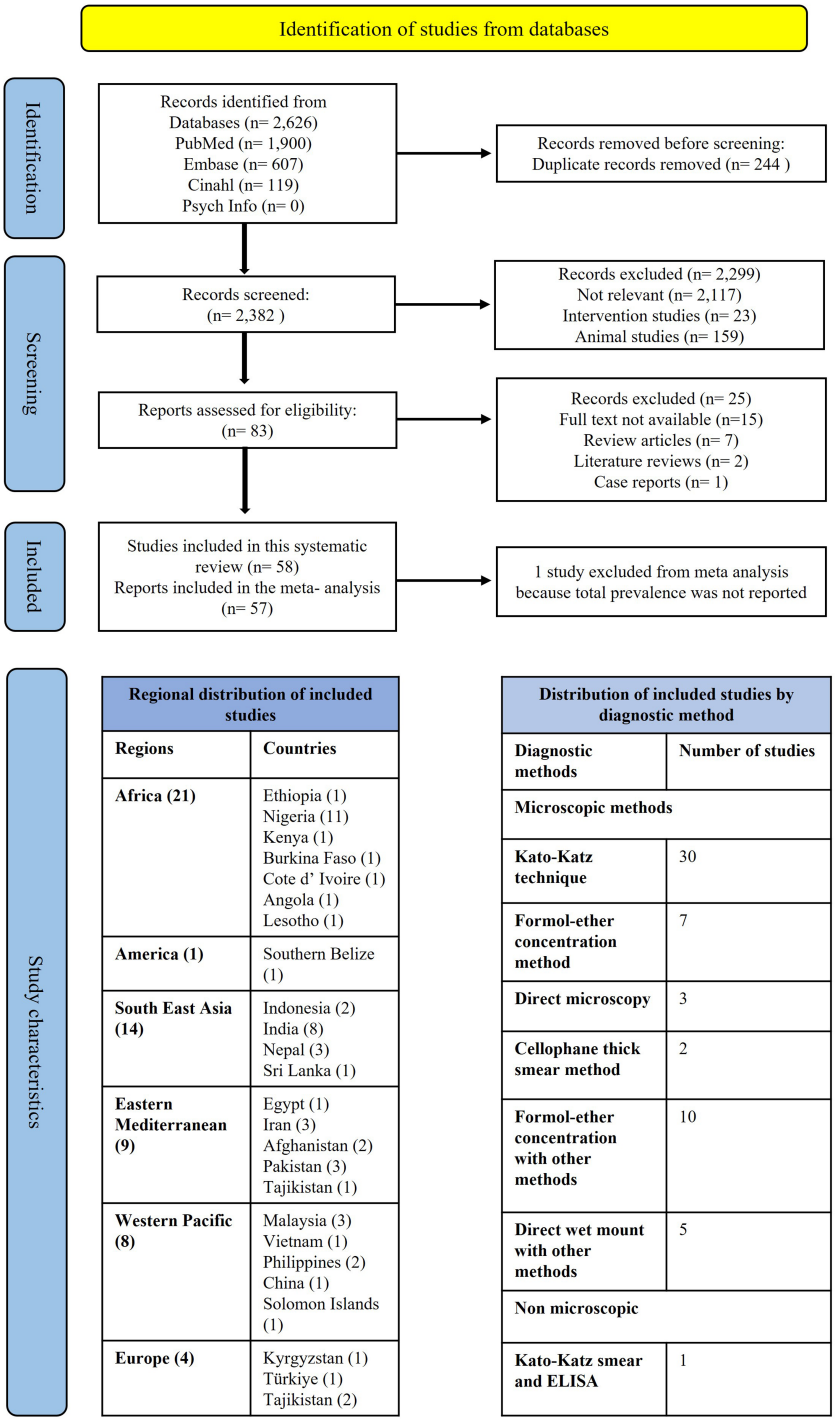
PRISMA flow diagram showing the selection of studies included in this systematic review.

Our initial search of electronic databases such as Medline via PubMed, Embase, Cinhal and Psychinfo yielded 2,626 articles, of which 2,382 records remained after removing duplications (244). Upon screening the articles, 2,299 articles were further excluded; 2,117 were irrelevant because they were of different study designs, had different outcomes, or had different study populations. A total of 23 studies were intervention studies about the sensitivity and specificity of STH diagnostics, and 159 articles were not about humans. Upon further assessment for eligibility, 7 studies that were review articles, 1 literature review and 2 case reports were excluded. After provisional screening of the title and abstract, 87 articles were included for full-text screening. Finally, 58 (21–72) published studies fulfilling the criteria were included in the final analysis (Table 1). The detailed list of included studies is given in Supplementary material S4. The PRISMA flow diagram for the selection of studies is given in Figure 1. The PRISMA checklist for this study is also given in the Supplementary material S5.

**Table 1:** Pooled prevalence of STH infection by region.

Variables	Number of studies	Sample	Cases	Prevalence (95% CI)	Heterogeneity		*p*-value
					Q	*I*^2^ (%)	
Overall prevalence	57	49,630	19,725	37.16 (29.74–44.89)	17346.5	99.7%	<0.001
Region							
Africa	21	14,027	6,056	37.10 (26.84–47.95)	3304.2	99.4%	<0.001
America	1	500	301	60.20 (55.76–64.52)	–	–	–
Southeast Asia	14	13,130	7,036	39.60 (25.67–54.41)	3098.36	99.6%	<0.001
Eastern Mediterranean	9	11,068	1,537	19.86 (8.05–35.30)	2535.70	99.7%	<0.001
Western Pacific	8	5,947	2,433	50.41 (33.74–67.04)	1116.3	99.4%	<0.0001
Europe	4	4,958	2,362	39.74 (20.40–61.0)	682.1	99.6%	<0.0001

### General characteristics of selected studies

The sample sizes of the included studies ranged from 139 (64) to 6,421 (43). A total of 48,782 children aged 5–18 were recruited for the studies. We categorized the regional distribution of our studies according to the World Health Organization’s classification of various countries into six regions. In total, 21 studies were from the African region (AFR) (21–35, 73–78), 1 study was from the American region (AMR) (36), 14 studies were from the Southeast Asia region (SER) (37–50), 4 studies were from the European region (EUR) (51–54), 9 studies were from the Eastern Mediterranean region (EMR) (55–63), and 9 studies were from the Western Pacific region (WPR) (64–72). All of our included studies were cross-sectional. The diagnostic methods used to detect STH infection in various studies include the Kato-Katz method (30), the Formol-ether concentration method (FEC) (7), the Cellophane thick smear (2), Direct microscopy (3), Formol-ether concentration with other methods (10), Direct wet mount with other methods (5), and the Kato-Katz smear with ELISA (1). A detailed description can be found in Figure 1. We assessed the quality of each study using the JBI critical appraisal tools and also reported the publication bias of the included studies using the funnel plot asymmetry in Supplementary material S2, S3.

### Pooled prevalence estimates of soil-transmitted helminths and heterogeneity

**Figure 2:**
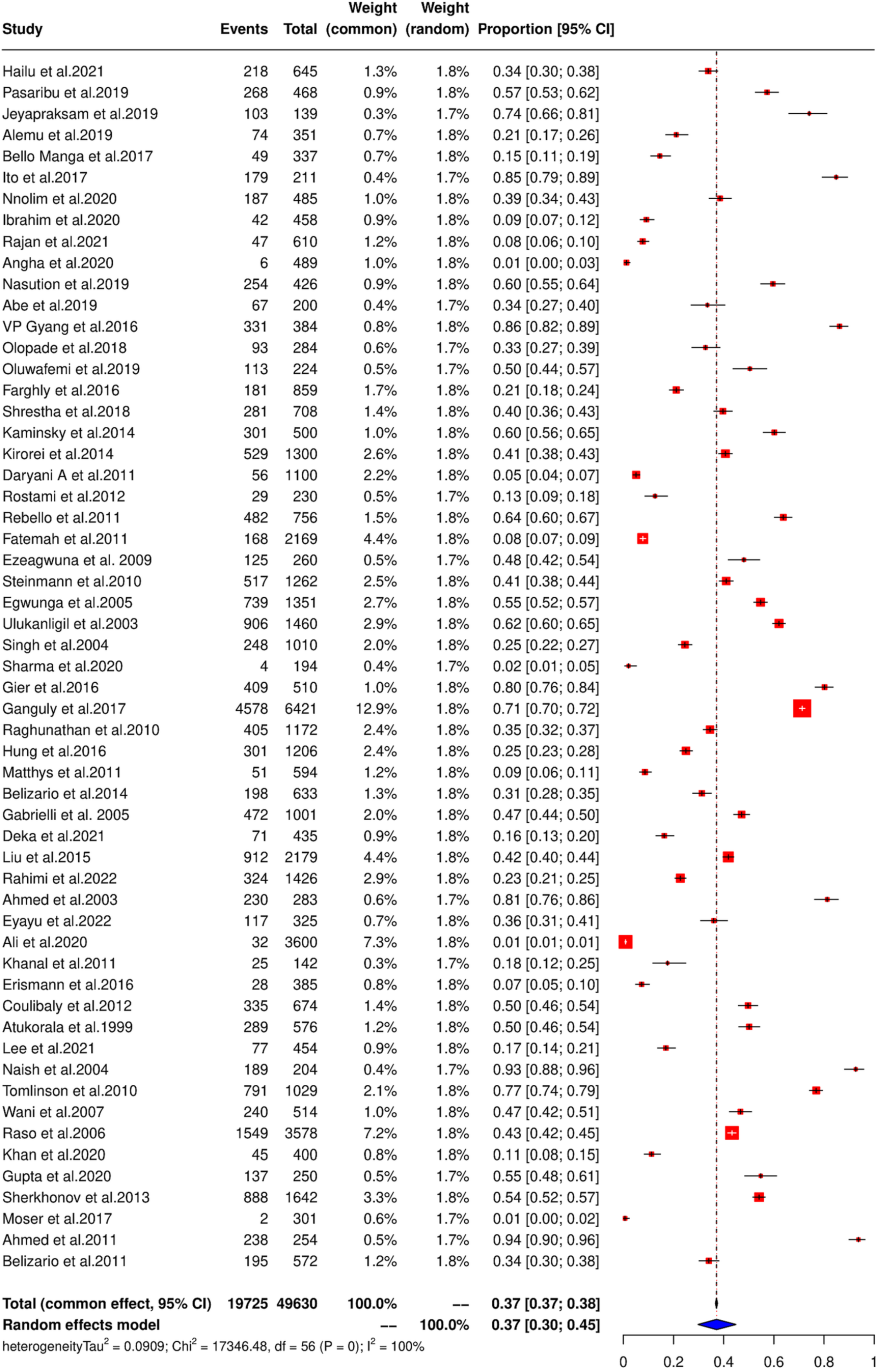
Pooled prevalence of STH among school-going children in LMICs.

Among a total of 57 studies from different regions, 49,630 school-going children (5–18 years) as study participants, the pooled prevalence and regional prevalence of STH infection were estimated. Among the total study participants, 19,725 children were found to be positive for STH infection, giving a pooled prevalence estimate of 37.16 (95% CI: 29.74–44.89) with considerable heterogeneity (χ2 = 17,346.5, *p* < 0.001; *I*^2^ = 99.70%) (Figure 2). The prevalence of STH was 37.10% (95% CI: 26.84–47.95) in Africa, 60.20% (95% CI: 55.76–64.52) in the America, 39.60% (95% CI: 25.67–54.41) in Southeast Asia, 22.87% (9.27–40.30) in the Eastern Mediterranean, 50.41% (95% CI: 33.74–67.04) in the Western Pacific and 35.10% (10.03–65.67) in the European region (Table 1). The forest plot showing the prevalence of STH in different regions is shown in Supplementary material S6.

### Pooled prevalence of species-specific soil-transmitted helminths

In the species-specific estimation of the pooled prevalence of various soil-transmitted helminths, the prevalence of *Ascaris lumbricoides* was found to be the highest [24.07% (95% CI: 17.07–31.83)] from the fifty-one included studies. From forty four studies, the prevalence of *Trichuris trichiura* was found to be 16.04% (95% CI: 10.92–21.91) followed by *hookworm* with a prevalence of 10.20 (95% CI: 6.63–14.41). The lowest prevalence was found for *Enterobius vermicularis* at 5.97% (95%CI: 1.56–12.90) from ten studies (Table 2).

**Table 2:** Species-specific pooled prevalence of STH infection.

Variables	Number of studies	Sample	Cases	Prevalence (95% CI)	Heterogeneity		*p*-value
					Q	*I*^2^ (%)	
*Ascaris lumbricoides*	51	38,972	10,965	24.07 (17.07–31.83)	14484.92	99.7%	<0.001
*Hookworm*	44	31,496	5,267	10.20 (6.63–14.41)	5349.50	99.2%	<0.001
*Trichuris trichiura*	44	30,536	4,474	16.04 (10.92–21.91)	7187.60	99.4%	<0.001
*Enterobius vermicularis*	10	8,848	840	5.97 (1.56–12.90)	1114.73	99.2%	<0.0001

### Subgroup analysis based on species of soil-transmitted helminths and sex by species

#### Pooled prevalence estimates of *Ascaris lumbricoides* and heterogeneity

Fifty-one studies consisting of 38,972 school-going children reported that the pooled prevalence of *Ascaris lumbricoides* was 24.07% (95% CI: 17.07–31.83) with substantial heterogeneity (χ2 = 14484.92, *p* < 0.001; *I*^2^ = 99.70%) (Supplementary material S7). The prevalence was 34.64% (95% CI: 21.16–50.0) in Southeast Asia, 28.20% (95% CI: 13.60–45.61) in Western pacific followed by 25% (95% CI: 14.70–37.0) in the African region. The detailed description is presented in Table 3.

**Table 3:** Regional subgroup analysis to estimate the pooled prevalence of different soil-transmitted helminths.

Regions	Number of studies	Sample	Cases	Prevalence (95% CI)	Heterogeneity		*p*-value
					Q	*I*^2^ (%)	
*Ascaris lumbricoides*							
Africa	17	8,040	2,223	25.0 (14.70–37.0)	2137.7	99.3%	<0.001
America	1	500	123	24.60 (20.90–28.62)	–	–	–
Southeast Asia	13	10,727	5,518	34.64 (21.16–50.0)	2010.20	99.4%	<0.001
Eastern Mediterranean	8	9,968	868	8.90 (1.70–20.70)	1861.30	99.6%	<0.001
Western Pacific	9	6,239	1,638	28.20 (13.60–45.61)	1546.7	99.5%	<0.0001
Europe	3	3,498	595	13.74 (5.53–24.80)	136.1	98.5%	<0.0001
*Hookworm*							
Africa	18	11,197	2,510	12.42 (5.43–21.70)	2699.2	99.4%	<0.001
America	1	500	110	22.0 (18.44–25.90)	–	–	–
Southeast Asia	11	9,909	1989	13.40 (7.20–21.10)	627.71	98.4%	<0.001
Eastern Mediterranean	4	3,057	148	3.40 (1.20–6.52)	43.13	93.0%	<0.001
Western Pacific	9	6,239	489	6.63 (1.97–13.70)	646.60	98.8%	<0.001
Europe	1	594	21	3.54 (2.20–5.35)	–	–	–
*Trichuris trichiura*							
Africa	15	7,148	1,198	10.80 (5.24–17.91)	1012.90	98.6%	<0.001
America	1	500	201	40.20 (35.90–44.65)	–	–	–
Southeast Asia	13	10,727	1,335	9.85 (9.30–10.43)	1614.60	99.3%	<0.001
Eastern Mediterranean	4	3,686	124	2.10 (0.10–7.10)	165.16	98.2%	<0.0001
Western Pacific	9	6,239	1,564	36.84 (15.80–61.0)	2782.10	99.7%	<0.001
Europe	2	2,236	52	2.05 (0.97–3.51)	3.63	72.4%	0.0569

#### Pooled prevalence estimates of *hookworm* and heterogeneity

Forty-four studies involving 31,496 children also reported the pooled prevalence of *hookworm*. Hence, the pooled prevalence of *hookworm* was 10.20% (6.63–14.41) with substantial heterogeneity (χ2 = 5349.50, *p* < 0.001; *I*^2^ = 99.20%) (Supplementary material S7). The prevalence of *hookworm* was 13.40% (95% CI: 7.20–21.10) in Southeast Asia, 12.42% (95% CI: 5.43–21.70) in Africa, followed by 6.63% (95% CI: 1.97–13.70) in Western Pacific region (Table 3).

#### Pooled prevalence estimates of *Trichuris trichiura* and heterogeneity

Forty-four studies involving 30,536 children reported that the pooled prevalence of *Trichuris trichiura* was 16.04% (95% CI: 10.92–21.91) with variable heterogeneity (χ2 = 7187.60, *p* < 0.001; *I*^2^ = 99.40%) (Supplementary material S7). The pooled prevalence of *Trichuris trichiura* was 36.84 (15.80–61.0) in the Western Pacific region, 10.80 (5.24–17.91) in the African region, 9.85 (9.30–10.43) in the Southeast Asia region followed by 2.21 (0.33–5.57) in the Eastern Mediterranean region (Table 3).

### Subgroup analysis based on sex

The prevalence of *Ascaris lumbricoides* was found to be higher in male subjects 28.75% (95% CI: 21.20–37.0) compared with female subjects 28.30% (95% CI: 20.90–36.34). A detailed description can be found in Table 4.

**Table 4:** Sex-specific subgroup analysis for estimation of the pooled prevalence of different soil-transmitted helminths.

Serial number	STH species	Prevalence		
		Overall prevalence	Prevalence among male subjects	Prevalence among female subjects
1.	*Ascaris lumbricoides*	24.07 (17.07–31.83)	28.75 (21.20–37.0)	28.30 (20.90–36.34)
2.	*Hookworm*	10.20 (6.63–14.41)	10.73 (6.70–15.55)	10.60 (6.64–15.30)
3.	*Trichuris trichiura*	16.04 (10.92–21.91)	17.44 (11.70–24.10)	17.44 (11.66–24.10)

### Regional distribution of eligible studies and risk zones for STH infections

The highest number of studies was from the African region with 21 (36.2%) and from Southeast Asia with 14 (24.1%) studies, respectively. These were followed by the Eastern Mediterranean and Western Pacific regions with 9 studies each (15.5%). Studies from Europe and the America were 4 (6.8%) and 1 (1.72%), respectively. The Western Pacific region is classified as a High-risk Zone (HRZ) with a prevalence of 50.41% according to the WHO risk classification (79). As the America contains only one study having STH of 60%, still we cannot classify it according to WHO risk classification (79). With STH prevalence values of 39.6, 37.1, 35.1, and 22.8%, respectively, Southeast Asia, Africa, Europe, and the Eastern Mediterranean area have been defined as moderate risk zones (MRZs). None of the regions were classified as low-risk zones (LRZs) based on the study findings.

### Trends of STH infection in different regions according to the included studies from 1999 to 2022

**Figure 3:**
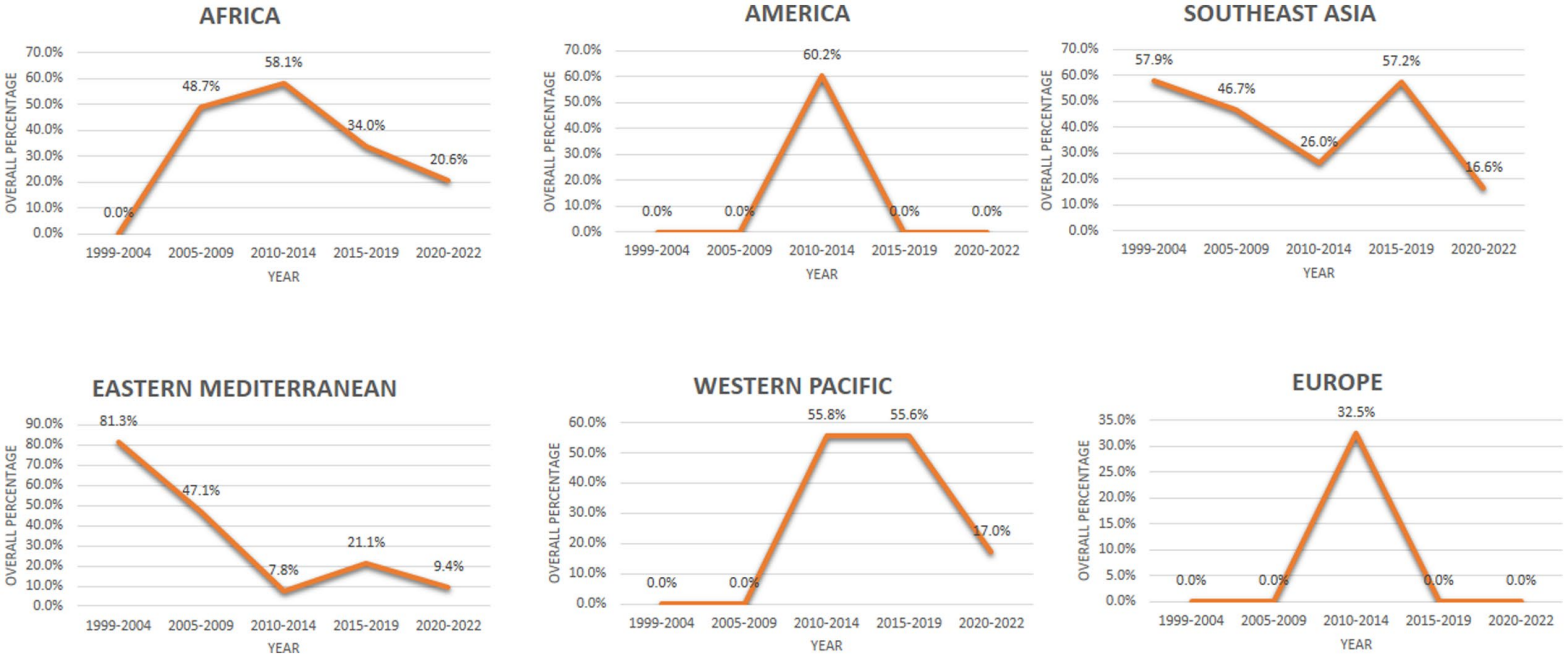
Regional trends of STH infection from 1999 to 2022.

To understand the trend, the time period was divided into two groups, i.e., (i) from 1999 to 2012 and, (ii) from 2013 to 2022. We observed that the overall prevalence of STH (soil-transmitted helminth) infection decreased by 12% from 1999 to 2012, from 44.61% (with a confidence interval of 33.90 to 55.61%) to 32.61% (with a confidence interval of 22.70 to 43.40%) in the years 2013–2022. The details of the included studies and the change in trend are shown in Table 5. The regional distribution of STH infection is shown in Figure 3.

**Table 5:** Subgroup analysis for pooled STH prevalence during the years 1999 to 2012 and 2013 to 2022.

Regions	Number of studies	Sample	Cases	Prevalence (95% CI)	Heterogeneity		*p*-value
					Q	*I*^2^ (%)	
Year of publication							
1999–2012	22	20,191	8,279	44.61 (33.90–55.61)	5150.9	99.6%	<0.001
2013–2022	35	29,439	11,446	32.61 (22.70–43.40)	12110.5	99.7%	<0.001

## Discussion

This study aimed to assess the prevalence of soil-transmitted helminths (STH) and identify associated factors among school-aged children. The results indicate a high overall pooled prevalence of STH, estimated at approximately 37.16% [95% CI: 29.74–44.89]. Additionally, the prevalence of STH showed regional differences, with the highest rates observed in the Western Pacific region (50.41%) and the lowest in the Eastern Mediterranean region (19.86%).

Species-specific analysis revealed that *Ascaris lumbricoides* was the most common STH, with a prevalence of 24.07%, followed by *Trichuris trichiura* at 16.04%. Notably, the Western Pacific region fell into the high-risk zone (HRZ) according to the World Health Organization’s risk classification for STH, while Southeast Asia, Africa, Europe, and the Eastern Mediterranean region fell into the moderate-risk zones (MRZs).

The primary purpose of conducting a systematic review and meta-analysis of the prevalence of soil-transmitted helminth infections, particularly among school-aged children, is to gain a thorough understanding of the disease burden in LMICs. This knowledge will aid in assessing the efficacy of current control and prevention strategies in these countries to control and eliminate these important NTDs. Furthermore, this study will help determine the benefits of various strategies aimed at managing and preventing soil-transmitted helminth infections and NTDs which in turn contribute to vivid outcomes like anemia, stunting, malnutrition, and developmental delays that further affect children’s education and their future prospects. Such policies could be further strengthened by supplementing national epidemiological data to maximize the impact of these diseases. STH infection is a major health concern in LMICs, and it has a direct or indirect impact on the population’s general health due to limited resources to address the problem.

The studies included in this systematic review offer helpful data that provides a bird’s-eye view of different regions organized according to the WHO regional classification from 1999 to 2022. Organizing and spatially distributing this information has the ability to help establish an integrated approach to STH infection control and prevention which requires the utmost urgency. The overall pooled prevalence of STH infection in LMICs is estimated at approximately 37.16% which is higher than systematic reviews conducted in Ethiopia 33 and 33.4%, respectively (80, 81). Some studies conducted in the Eastern Mediterranean (9.48%) (82) and South America (27.1%) (83), reported lower rates of STH prevalence than the present study. However, studies from Sub-Saharan regions such as Nigeria reported a prevalence of about 54.8%, which is higher than this study (84). Differences in results could be due to differences in the sensitivity and specificity of the diagnostics used, and climatic conditions (such as moisture content, precipitation, and temperature). Furthermore, WASH (Water, Sanitation and Health) indicators among study participants and the number of studies completed during this period may have contributed to these discrepancies. Also, we only focused on prevalence studies that detected all cases, avoiding studies that took into account ongoing Mass Drug Administration (MDA) protocols and the provision of anthelminthic drugs to identified cases.

The WHO regional classification was used to estimate the burden of STHs. The present study revealed that STH infections were more prevalent in the Western Pacific followed by Europe and Southeast Asia regions and that they were not distributed uniformly across the globe. The majority of these STHs are exacerbated by poor living conditions, low socioeconomic levels, poor personal hygiene, and insufficient environmental sanitation. These conditions can increase the likelihood of exposure to infectious agents and could contribute to an environment conducive to disease transmission and persistence, particularly in remote and high-risk areas.

The time trend analysis showed a significant decline in the overall prevalence over time from 1999 to 2022 There are large regional variations in the trend of decline in STH cases on an annual basis. This variation could be attributed to a multitude of factors including increased awareness and adoption of hygienic practices, coordinated deworming programs at the national level, and the implementation of educational campaigns. Although the prevalence of STHs has decreased, this drop has been relatively slow at almost 10% with the prevalence falling from 44.61% in 1999–2012 to 32.61% in 2013–2022. This may be related to increasing population estimates in LMICs as well as the widespread use of a single treatment regimen for deworming, which may lead to drug-resistant strains of STHs, resulting in a slower rate of decline in the prevalence of STHs (85, 86).

*Ascaris lumbricoides* was found to be the most common (24.07%) form of STH in the present study. This finding is consistent with a study conducted in Sri Lanka (87), India (88), Malaysia (89), and Cuba (90), respectively where the prevalence of *A. lumbricoides* was found to be highest. On the other hand, higher prevalence rates were observed in Nigeria (44.6%), Rwanda (38.6%), Uganda (43.5%) (91), and Kenya (24.3%) (92) than in the present study. School-going children tend to spend their leisure time playing outdoors with sand and eating unclean food leftovers under trees with unwashed hands. This ultimately keeps them at a higher risk of exposure to the infective stage of Ascaris parasites compared to preschool children (87). Similarly, the pooled prevalence of *hookworm*, followed by *A. lumbricoides,* was 10.20%. Specifically, the prevalence found in the current study was lower than the prevalence reported in previous studies conducted in Uganda (91) and Nigeria (84) and higher than in studies conducted in Cameroon (93) and Rwanda (94). The third most common species was *Trichuris trichiura* with a pooled prevalence estimated at 16.04%. The results of this study show that the observed prevalence is significantly higher than in studies from Malaysia (2.7%) (95), China (2.4%) (96), and Bangladesh (10.52%) (97). The variation in species between different regions can be attributed to a multitude of factors, including environmental permissibility, geographic distribution, agricultural practices, precipitation levels, and climatic variations, among others. These factors may contribute to the prevalence of a particular species in one region over another. Interestingly, it is possible for multiple species to coexist within a single region, resulting in mixed infections in the general population.

At least 120 countries are affected by endemic STH, accounting for more than 5 million DALYs to date (98). Nonetheless, the number of DALYs caused by STH has decreased by 3.5 million as a result of the global rise in the use of preventative chemotherapy treatments for STH management, which went from 5 to 60% between 2000 and 2019. Despite continuous efforts to bring awareness and curb the incidence of new cases the countries preferably with low resource settings and urban poor are still under the vicious clutch of helminth infection. To effectively reduce STH infections, several challenges must be tackled, including the potential for anthelmintic drug resistance, inadequate diagnosis during program implementation, the limited effectiveness of existing treatments, and shortages in the pharmaceutical supply (99). To address these challenges, it is critical to develop novel STH control targets and tactics that go beyond the existing practice of delivering anthelmintic medications only to schoolchildren. This may involve creating novel diagnostic tools to detect high-risk individuals and tailoring interventions to the specific requirements of different communities.

Furthermore, encouraging responsible use of anthelmintic drugs and exploring alternative therapies that are less susceptible to resistance can aid in the fight against drug resistance. We conducted an in-depth search of numerous databases and sources to discover relevant papers on STH-infected populations. Our study offers insightful data to assist policymakers in deciding whether to strengthen or change current control and preventative measures. In order to support international efforts to eradicate STH, we selected geographic regions at high risk of STH infection that should be prioritized for MDA and other interventions. To reduce the burden of STH and other parasite diseases, it is critical to prioritize these areas and ensure the implementation of appropriate management methods. Furthermore, because there is an urgent need to enhance the accuracy of STH diagnosis and management, we advocate for large-scale research employing sensitive diagnostic methods on repeated stool samples to measure the degree of STH infection in children.

### Strengths and limitations

This systematic review is a groundbreaking effort to better understand STH infection among school-aged children in low- and middle-income countries. This is the first complete assessment of the overall burden of STH infection in this population, and it takes into account regional helminth infestation prevalence, sex-specific prevalence, and age group prevalence. This systematic review includes all cross-sectional studies of STH infection in school-going children in LMICs, which is a major strength in itself. Additionally, this study sheds light on the prevalence of helminth infection in different locations, providing a more comprehensive picture of helminth occurrence. This information is critical for the creation of species-specific anti-infection targets. Our study also has certain limitations. First, the inclusion and exclusion criteria necessarily resulted in some publication and translation bias. Publication bias refers to the tendency of journals to publish positive or statistically significant results more often than negative or null findings, potentially skewing the available literature. Translation bias occurs when research from one language or region is disproportionately represented, potentially leading to cultural or regional specificity in the findings. It is critical to address the extent and potential magnitude of these biases in order to accurately assess their generalizability and reliability. Without a thorough understanding, the validity and applicability of the study findings may be compromised, ultimately affecting the ability to make informed decisions and draw accurate conclusions. Some studies that measured moderate prevalence were excluded from meta-analyses due to insufficient data on the total number of participants and/or the number of participants by sex. Several studies have used the Kato-Katz diagnostic test; however, more precise diagnostic procedures, particularly those based on multi-parallel quantitative polymerase chain reaction (qPCR), are essential for lower-prevalence settings and lower-intensity investigations (100). Despite the substantial risk of bias, this review identified all the cross-sectional studies that still offer useful information and could be more widely used in scientific research.

While we used the JBI checklist to evaluate the quality of the studies, it is clear that there is a wide range in how well these cross-sectional studies adhere to rigorous methodology. The JBI checklist does not rely on fixed scores, which means we can look at the bigger picture. After a thorough evaluation, we found that some studies closely aligned with the checklist criteria, while others fell short in important aspects. These differences in methodological quality highlight the potential for bias in the cross-sectional studies we reviewed. It is important to keep this potential bias in mind when interpreting the results and implications of this review. Despite these methodological challenges, it is worth noting that these studies still offer valuable insights, and although we need to interpret their findings carefully, they remain relevant and useful in the field of scientific research.

The overall prevalence was 37% which is still a high prevalence of STH across school-going children in LMICs. DALYs due to soil-transmitted helminthiases (STH), which are related to both the severity of the infection and the source of the disability along with nutritional deficiency and growth retardation. This decreases the quality of life in places with few resources and a high risk of transmission alongside increasing the DALY. Albendazole MDA and the WASH technique have been proven to be important controlling factors in the prevention of STH, but in retrospect, a progressive approach is needed to reduce the prevalence.

### Future implications

While the administration of MDA has a major positive impact on children, there is still a scarcity of data on the completion of deworming treatment among school-going children. Incomplete administration further leads to helminthic resistance, which contributes to other health problems that will be difficult to treat in the future. Monitoring of deworming among young female children will help keep STH-induced anemia under control along with Weekly Iron Folic Acid Supplementation (WIFS). This will not only prevent anemia but also reduce complications associated with anemia in the reproductive years. Regions with high levels of helminthic infection still lack extensive longitudinal studies, which may lead to an evidence gap and hamper burden estimation. Continuous assessment of high-burden areas and rampant evaluation can provide an overview of the overall situation and help focus on preventive treatment.

## Data availability statement

The original contributions presented in the study are included in the article/Supplementary material, further inquiries can be directed to the corresponding authors.

## Author contributions

RA: Conceptualization, Data curation, Formal analysis, Methodology, Software, Visualization, Writing – original draft, Writing – review & editing. SwP: Conceptualization, Data curation, Formal analysis, Methodology, Software, Visualization, Writing – original draft, Writing – review & editing. JSK: Conceptualization, Data curation, Investigation, Resources, Software, Supervision, Validation, Visualization, Writing – review & editing. SK: Methodology, Supervision, Validation, Visualization, Writing – review & editing. NM: Methodology, Supervision, Validation, Visualization, Writing – review & editing. SuP: Conceptualization, Data curation, Formal analysis, Funding acquisition, Investigation, Methodology, Resources, Supervision, Visualization, Writing – review & editing. SaP: Conceptualization, Data curation, Funding acquisition, Methodology, Resources, Software, Supervision, Validation, Visualization, Writing – review & editing.

## Glossary

AFR: African region
AMR: American region
DALYs: Disability-Adjusted Life Years
EMR: Eastern Mediterranean region
EUR: European region
FEC: Formol-ether concentration method
GBD: Global Burden of Disease 2019
HRZs: High-risk Zones
LMIC: Low- and Middle-Income Countries
LRZs: low-risk zones
MDA: Mass Drug Administration
MeSH: Medical Subject Heading
MRZs: Moderate risk zones
NTDs: neglected tropical diseases
PRISMA: Preferred Reporting Items for Systematic reviews and Meta-analyses
PSAC: Preschool aged children
SAC: School aged children
SER: Southeast Asia region
STH: Soil-transmitted helminths
RZs: Risk zones
WASH: Water, Sanitation and Health
WHO: World Health Organization
WPR: Western Pacific region
WIFS: Weekly Iron Folic Acid Supplementation
