# Supplementary material for: Prevalence and correlates of soil-transmitted helminths in schoolchildren aged 5 to 18 years in low- and middle-income countries: a systematic review and meta-analysis
Source: Front Public Health. 2024 Mar 21;12:1283054. doi: 10.3389/fpubh.2024.1283054 (PMC10991833; doi:10.3389/fpubh.2024.1283054)
Supplement: Supplementary file 1 [file Table_1.docx]

**Search strategy for soil transmitted helminthes among school going children in LMICs**

[1] (((((((((((((((((((((((((((((((((((((((((((((((((((((((((((((((((LMIC[Title/Abstract]) OR (low middle income countr*[Title/Abstract])) OR (Angola[Title/Abstract])) OR (Algeria[Title/Abstract])) OR (Bangladesh[Title/Abstract])) OR (Belize[Title/Abstract])) OR (Bhutan[Title/Abstract])) OR (Bolivia[Title/Abstract])) OR (Cabo Verde[Title/Abstract])) OR (Cambodia[Title/Abstract])) OR (Cameroon[Title/Abstract])) OR (Congo Republic[Title/Abstract])) OR (Cote d'Ivoire[Title/Abstract])) OR (Djibouti[Title/Abstract])) OR (Egypt[Title/Abstract])) OR (Arab Republic[Title/Abstract])) OR (El Salvador[Title/Abstract])) OR (Eswatini[Title/Abstract])) OR (Ghana[Title/Abstract])) OR (Haiti[Title/Abstract])) OR (Honduras[Title/Abstract])) OR (India[Title/Abstract])) OR (Indonesia[Title/Abstract])) OR (Iran[Title/Abstract])) OR (Islamic Republic[Title/Abstract])) OR (Kenya[Title/Abstract])) OR (Kiribati[Title/Abstract])) OR (Kyrgyz Republic[Title/Abstract])) OR (Lao PDR[Title/Abstract])) OR (Lesotho[Title/Abstract])) OR (Mauritania[Title/Abstract])) OR (Micronesia[Title/Abstract])) OR (Fed sts[Title/Abstract])) OR (Mongolia[Title/Abstract])) OR (Morocco[Title/Abstract])) OR (Myanmar[Title/Abstract])) OR (Nepal[Title/Abstract])) OR (Nicaragua[Title/Abstract])) OR (Nigeria[Title/Abstract])) OR (Pakistan[Title/Abstract])) OR (Papua New Guinea[Title/Abstract])) OR (Philippines[Title/Abstract])) OR (Samoa[Title/Abstract])) OR (Sao tome[Title/Abstract] AND principe[Title/Abstract])) OR (Senegal[Title/Abstract])) OR (Solomon Island[Title/Abstract])) OR (Sri Lanka[Title/Abstract])) OR (Tanzania[Title/Abstract])) OR (Tajikistan[Title/Abstract])) OR (Timor Leste[Title/Abstract])) OR (Tunisia[Title/Abstract])) OR (Ukraine[Title/Abstract])) OR (Uzbekistan[Title/Abstract])) OR (Vanuatu[Title/Abstract])) OR (Vietnam[Title/Abstract])) OR (West bank[Title/Abstract] AND Gaza[Title/Abstract])) OR (Zambia[Title/Abstract])) OR (Zimbabwe[Title/Abstract])) OR (middle income[Title/Abstract])) OR (resource poor[Title/Abstract])) OR (global south[Title/Abstract])) OR (LMC[Title/Abstract])) OR (Resource poor[Title/Abstract])) OR (Developing countr*[MeSH Terms])) OR (developing nation[MeSH Terms])) OR (Low income population*[MeSH Terms])

[2] ((((((((Prevalence stud*[MeSH Terms]) OR (prevalence*[MeSH Terms])) OR (cross sectional stud*[MeSH Terms])) OR (cross sectional analys*[MeSH Terms])) OR (cross sectional survey*[MeSH Terms])) OR (risk factor*[MeSH Terms]))) OR (observational study[Title/Abstract])) OR (survey[Title/Abstract])

[3] (((((((((((children[Title/Abstract]) OR (childhood[Title/Abstract]))) OR (school age*[Title/Abstract])) OR (Schooler[Title/Abstract])) OR (Preadolescen*[Title/Abstract])) OR (school going children[Title/Abstract])) OR (aged 5-18 year*[Title/Abstract])) OR (child[MeSH Terms])) OR (children[MeSH Terms])) OR (adolescen*[MeSH Terms])) OR (School age population*[MeSH Terms])

[4] ((((((((((((((((((STH[Title/Abstract]) OR (Soil transmitted helminth*[Title/Abstract])) OR (Geo helminth*[Title/Abstract])) OR (helminth[MeSH Terms])) OR (ascaris lumbricoide*[MeSH Terms])) OR (stercorali*, strongyloide*[MeSH Terms])) OR (trichurias*[MeSH Terms])) OR (necator americanus[MeSH Terms])) OR (animal helminthias*[MeSH Terms])) OR (Cestode infection*[MeSH Terms])) OR (Enterobias*[MeSH Terms])) OR (Trematod*[MeSH Terms])) OR (threadworm[MeSH Terms])) OR (hookworm[MeSH Terms])) OR (hookworm infection*[MeSH Terms])) OR (roundworm[Title/Abstract])) OR (whipworm[Title/Abstract])) OR (trichuris trichiura[MeSH Terms]) ) OR (ancylostoma duodenale[MeSH Terms])
