# Supplementary material for: Prevalence and correlates of soil-transmitted helminths in schoolchildren aged 5 to 18 years in low- and middle-income countries: a systematic review and meta-analysis
Source: Front Public Health. 2024 Mar 21;12:1283054. doi: 10.3389/fpubh.2024.1283054 (PMC10991833; doi:10.3389/fpubh.2024.1283054)
Supplement: Supplementary file 2 [file Table_2.docx]

|  | **INCLUSION CRITERIA** | **STUDY SUBJECT AND SETTING** | **EXPOSURE MEASUREMENT** | **OBJECTIVE AND STANDARD CRITERIA USED FOR MEASURENEMT** | **CONFOUNDING IDENTIFIED** | **STRETEGIES TO DEAL WITH CONFOUNDING** | **OUTCOME MEASUREMENT** | **APPROPRIATENESS OF STATISTICAL ANALYSIS** | **QUALITY** |
| --- | --- | --- | --- | --- | --- | --- | --- | --- | --- |
| Hailu et al.2021 | Y | Y | Y | Y | Y | Y | Y | Y | 8 |
| Pasaribu et al. 2019 | Y | Y | Y | Y | N | N | Y | Y | 6 |
| Jeyaprakasam et al.2019 | N | Y | N | Y | N | N | Y | N | 3 |
| Alemu et al. 2019 | Y | Y | Y | Y | N | N | Y | Y | 6 |
| Bello manga et al. 2017 | Y | Y | Y | Y | N | N | Y | N | 5 |
| Ito et al. 2017 | N | Y | N | Y | N | N | Y | N | 3 |
| Nnolim et al.2020 | Y | Y | Y | Y | Y | Y | Y | Y | 8 |
| Ibrahim et al. 2020 | Y | Y | N | Y | N | N | Y | N | 4 |
| Rajan et al. 2020 | Y | Y | Y | Y | N | N | Y | Y | 6 |
| Anagha et al. 2020 | Y | Y | Y | Y | N | N | Y | Y | 6 |
| Nasution et al. 2019 | N | Y | Y | Y | N | N | Y | Y | 5 |
| Abe et al. 2019 | N | Y | N | Y | N | N | Y | Y | 4 |
| Gyang et al. 2019 | N | Y | N | Y | N | N | Y | Y | 4 |
| Olopade et al. 2018 | N | Y | Y | Y | N | N | Y | Y | 5 |
| Oluwafemi et al. 2019 | Y | Y | N | Y | N | N | Y | Y | 5 |
| Farghly et al. 2016 | Y | Y | N | Y | N | N | Y | Y | 5 |
| Shrestha et al. 2015 | Y | Y | N | Y | N | N | Y | Y | 5 |
| Kaminsky et al. 2014 | Y | Y | N | Y | N | N | Y | Y | 5 |
| Kirorei et al. 2014 | Y | Y | N | Y | N | N | Y | Y | 5 |
| Daryani et al.2011 | N | Y | N | Y | N | N | Y | Y | 4 |
| Rostami et al. 2012 | Y | Y | N | Y | N | N | Y | Y | 5 |
| Rebello et al. 2011 | Y | Y | Y | Y | N | N | Y | Y | 6 |
| Fatemah et al. 2011 | Y | Y | N | Y | N | N | Y | Y | 5 |
| Ezeagwuna et al. 2009 | N | Y | Y | Y | N | N | Y | Y | 5 |
| Steinmann et al. 2010 | N | Y | Y | Y | N | N | Y | Y | 5 |
| Egwunga et al. 2005 | Y | Y | N | Y | N | N | Y | Y | 5 |
| Ulukanligil et al. 2003 | Y | Y | N | Y | N | N | Y | Y | 5 |
| Singh et al. 2004 | Y | Y | N | Y | N | N | Y | Y | 5 |
| Sharma et al. 2020 | Y | Y | N | Y | N | N | Y | N | 4 |
| Gier et al. 2016 | Y | Y | Y | Y | N | N | Y | Y | 6 |
| Ganguly et al. 2015 | Y | Y | Y | Y | N | N | Y | Y | 6 |
| Raghunathan et al. 2010 | Y | Y | Y | Y | N | N | Y | Y | 6 |
| Hung et al. 2016 | N | N | N | Y | N | N | Y | Y | 3 |
| Matthys et al. 2011 | Y | Y | Y | Y | N | N | Y | Y | 6 |
| Belizario et al. 2014 | Y | Y | N | Y | N | N | Y | Y | 5 |
| Gabrielli et al. 2005 | Y | Y | N | Y | N | N | N | N | 3 |
| Deka et al. 2021 | Y | Y | N | Y | N | N | Y | Y | 5 |
| Liu et al. 2015 | Y | Y | N | Y | N | N | Y | Y | 5 |
| Rahimi et al. 2022 | Y | Y | N | Y | N | N | Y | Y | 5 |
| Ahmed et al. 2014 | Y | Y | N | Y | N | N | Y | N | 4 |
| Eyayu et al. 2022 | N | Y | Y | Y | Y | Y | Y | Y | 7 |
| Ali et al. 2020 | N | Y | N | Y | N | N | Y | Y | 4 |
| Khanal et al. 2011 | N | Y | N | Y | N | N | Y | Y | 4 |
| Mekhlafi et al. 2007 | Y | Y | Y | Y | N | N | Y | Y | 6 |
| Erismann et al. 2016 | Y | Y | Y | Y | N | N | Y | Y | 6 |
| Coulibaly et al. 2012 | Y | Y | N | Y | N | N | Y | Y | 5 |
| Atukorala et al. 1999 | Y | Y | Y | Y | N | N | Y | Y | 6 |
| Lee et al. 2021 | Y | Y | N | N | N | N | Y | N | 3 |
| Naish et al. 2004 | Y | Y | N | Y | N | N | Y | N | 4 |
| Tomlison et al. 2010 | Y | Y | N | Y | N | N | Y | Y | 5 |
| Wani et al. 2007 | Y | Y | N | Y | N | N | Y | Y | 5 |
| Raso et al. 2006 | Y | Y | Y | Y | N | N | Y | Y | 6 |
| Khan et al. 2020 | Y | Y | Y | Y | N | N | Y | Y | 6 |
| Gupta et al. 2020 | Y | Y | Y | Y | N | N | Y | Y | 6 |
| Sherkhonov et al. 2013 | Y | Y | Y | Y | N | N | Y | Y | 6 |
| Moser et al. 2017 | Y | Y | N | Y | N | N | Y | Y | 5 |
| Ahmed et al. 2011 | Y | Y | Y | Y | Y | Y | Y | Y | 8 |
| Belizario et al. 2011 | Y | Y | N | Y | N | N | Y | Y | 5 |
| Shrestha et al. 2018 | Y | Y | Y | Y | Y | Y | Y | Y | 8 |
