# Supplementary figures and images for: Prevalence and correlates of soil-transmitted helminths in schoolchildren aged 5 to 18 years in low- and middle-income countries: a systematic review and meta-analysis
Source: Front Public Health. 2024 Mar 21;12:1283054. doi: 10.3389/fpubh.2024.1283054 (PMC10991833; doi:10.3389/fpubh.2024.1283054)

**<Supplementary file:3> Publication bias assessment plot: Funnel plot and Begg’s corelation test**


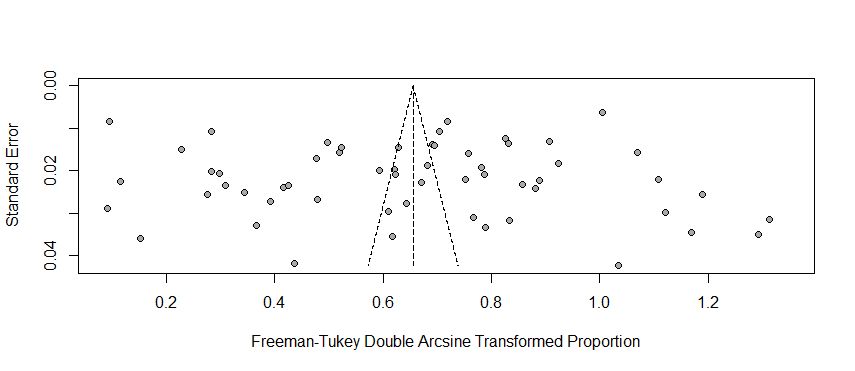

Supplement: Supplementary file 3 [file Table_3.docx]
