# Supplementary material for: Prevalence and correlates of soil-transmitted helminths in schoolchildren aged 5 to 18 years in low- and middle-income countries: a systematic review and meta-analysis
Source: Front Public Health. 2024 Mar 21;12:1283054. doi: 10.3389/fpubh.2024.1283054 (PMC10991833; doi:10.3389/fpubh.2024.1283054)
Supplement: Supplementary file 4 [file Table_4.docx]

**Supplementary file 4**

**List of included studies for systematic reviews**

| **REGIONS** | **STUDY ID** | **COUNTRY** | **STUDY SETTING** | **STUDY DESIGN** | **AGE GROUP** | **SAMPLE SIZE** | **MALE** | **FEMALE** | **LAB METHOD** |
| --- | --- | --- | --- | --- | --- | --- | --- | --- | --- |
| **AFRICAN**  **REGION**  **(AFR)** | Hailu et al.2021 [22] | Ethiopia | Primary school children | Cross-sectional study | 6-15 years | 614 | 308 | 337 | Direct smear and formol ether concentration |
|  | Alemu et al.2019 [23] | Ethiopia | Primary school children | Cross-sectional study | 5-14 years | 351 | 180 | 171 | Direct saline and iodine wet mount |
|  | Bello Manga et al.2017 [24] | Nigeria | Grazing reserve | Cross-sectional study | 5-15 years | 340 | 150 | 190 | Formol-ether concentration method |
|  | Ito et al.2017 [25] | Nigeria | Primary school children | Cross-sectional study | 5-13 years | 211 | 112 | 99 | Kato-Katz smear |
|  | Nnolim et al.2020 [26] | Nigeria | Primary school children | Cross-sectional study | 5-16 years | 485 | 229 | 256 | Formolether concentration method |
|  | Ibrahim et al.2020 [27] | Nigeria | Primary school children | Cross-sectional study | 5-18 years | 458 | NA | NA | Kato-Katz smear |
|  | Angha et al.2020 [28] | Nigeria | Secondary school | Cross-sectional study | 10-17 years | 489 | 261 | 228 | Wet mount and formolether concentration method |
|  | Abe et al.2019 [29] | Nigeria | Primary school children | Cross-sectional study | 5-16 years | 200 | 80 | 120 | Cellophane thick smear method |
|  | VP Gyang et al.2016 [30] | Nigeria | Primary school children | Cross-sectional study | 7-17 years | 384 | 187 | 195 | Kato-Katz smear |
|  | Olopade et al.2018 [31] | Nigeria | Primary school children | Cross-sectional study | 6-12 years | 384 | 190 | 194 | Saline iodine mount and formolether concentration method |
|  | Oluwafemi et al.2019 [32] | Nigeria | Primary school children | Cross-sectional study | 5-15 years | 224 | 113 | 111 | Direct wet mount and formol-ether concentration method |
|  | Kirorei et al.2014 [33] | Kenya | Primary school children | Cross-sectional study | 5-12 years | 1300 | NA | NA | Kato-Katz smear |
|  | Rebello et al.2011 [34] | Southern Cameroon | Rural schools | Cross-sectional study | 5-12 years |  | NA | NA | Direct smear and formol-ether concentration method |
|  | Ezeagwuna et al.2010 [35] | Nigeria | Public school children’s | Cross-sectional study | 5-16 years | 260 | 123 | 137 | Formolether concentration method |
|  | Egwunyenga et al.2005 [36] | Nigeria | Government schools | Cross-sectional study | 5-15 years | 1351 | 740 | 611 | Kato-Katz smear |
|  | Eyayu et al.2022 [37] | Ethiopia | Elementary schools | Cross-sectional study | 6-13 years | 325 | 145 | 179 | Kato Katz technique |
|  | Eismann et al.2016 [38] | Burkina Faso | Primary school children | Cross-sectional study | 8-14 years | 385 | 197 | 188 | Kato Katz technique |
|  | Coulibaly et al.2012 [39] | Cote d'Ivoire | Primary school children | Cross-sectional study | 8-12 years | 674 | 343 | 331 | Kato Katz technique |
|  | Tomlinson et al.2010 [40] | Angola | Primary school children | Cross-sectional study | 6-10 years | 1029 | 516 | 513 | Direct microscopy |
|  | Raso et al.2006 [41] | Cote d'Ivoire | School children in rural areas | Cross-sectional study | 6-16 years | 3578 | 2162 | 1416 | Kato Katz technique |
|  | Moser et al.2017 [42] | Lesotho | Primary school children | Cross-sectional study | 8-14 years | 301 | 139 | 162 | Kato Katz technique |
| **REGION OF AMERICA (AMR)** |  | | | | | | | | |
|  | Kaminsky et al.2014 [43] | Southern Belize | Rural and urban schools | Cross-sectional study | 5-12 years | 495 | 220 | 275 | Kato-Katz smear |
| **SOUTH EAST ASIA REGION (SEAR)** |  | | | | | | | | |
|  | Pasaribu et al.2019 [44] | Indonesia | Primary school children | Cross-sectional study | 6-12 years | 468 | 239 | 229 | Kato-Katz smear |
|  | Rajan et al.2021 [45] | India | Rural health training centre | Cross-sectional study | 5-14 years | 610 | 292 | 318 | Formolether concentration method |
|  | Nasution et al.2019 [46] | Indonesia | Primary school children | Cross-sectional study | 6-10 years | 426 | 229 | 197 | Kato-Katz smear |
|  | Shrestha A et al.2018 [47] | Nepal | Schools | Cross-sectional study | 8-15 years | 708 | 339 | 369 | Kato-katz smear |
|  | Singh et al.2004 [48] | India | Urban and rural schools | Cross-sectional study | 5-10 years | 1010 | 552 | 458 | Wet film and formolether concentration method |
|  | Sharma et al.2020 [49] | Nepal | Public school childrens | Cross-sectional study | 6-14 years | 194 | 113 | 81 | Direct wet mount method |
|  | Ganguly et al.2017 [50] | India | Government primary schools | Cross-sectional study | 5-10 years | 6421 | 3212 | 3209 | Kato Katz technique |
|  | Raghunathan et al.2010 [51] | India | Public school childrens | Cross-sectional study | 5-10 years | 1172 | 674 | 498 | Formolether concentration and saline wet mount method |
|  | Deka et al.2021[52] | India | Primary school children | Cross-sectional study | 5-13 years | 560 | 238 | 197 | Kato Katz technique |
|  | Khanal et al.2011 [53] | Nepal | High school children | Cross-sectional study | 6-16 years | 142 | 68 | 74 | Formolether concentration method |
|  | Atukorala et al.1999 [54] | Sri lanka | School children | Cross-sectional study | 14-18 years | 576 | NA | 576 | Kato Katz technique |
|  | Naish et al.2004 [55] | India | Primary school children | Cross-sectional study | 5-9 years | 235 | 103 | 101 | Formal-ether concentration method |
|  | Wani et al.2007 [56] | India | Middle school children | Cross-sectional study | 5-14 years | 514 | 266 | 248 | Direct microscopy |
|  | Gupta et al.2020 [57] | India | Urban resettlement |  | 6-14 years | 250 | 104 | 146 | Kato Katz technique |
|  | | | | | | | | | |
| **EUROPEAN REGION (EUR)** | Steinmann et al.2010 [58] | Kyrgyzstan | Primary school children | Cross-sectional study | 6-15 years | 1262 | 595 | 665 | Kato-Katz smear |
|  | Ulukanligil et al.2003 [59] | Turkey | Primary school children | Cross-sectional study | 7-14 years | 1820 | 1120 | 700 | Cellophane thick smear method |
|  | Matthys et al.2011 [60] | Tajikistan | Primary school children | Cross-sectional study | 7-11 years | 594 | 307 | 287 | Kato Katz technique |
|  | Sherkhonov et al.2013 [61] | Tajikistan | School children | Cross-sectional study | 7-11 years | 1642 | 841 | 801 | Kato Katz technique |
|  | | | | | | | | | |
| **EASTERN MEDITERRIAN REGION (EMR)** | Farghly et al.2016 [62] | Egypt | Primary school children | Cross-sectional study | 6-13 years | 859 | NA | NA | Formolether concentration method |
|  | Daryani A et al.2011 [63] | Iran | Primary and secondary school children | Cross-sectional study | 7-14 years | 1100 | 607 | 493 | Direct wet mount and formol-ether concentration method |
|  | Rostami et al.2012 [64] | Iran | Primary school children | Cross-sectional study | 7-12 years | 800 | NA | NA | Direct smear and formol-ether concentration method |
|  | Fatemah et al.2011 [65] | Iran | Elementary schools | Cross-sectional study | 6-11 years | 2169 | 996 | 1173 | Direct wet and formalin-ethyl acetate method |
|  | Gabrielli et al. 2005 [66] | Afghanistan | Primary school children | Cross-sectional study | 8-15 years | 1001 | 553 | 447 | Kato Katz technique |
|  | Rahimi et al.2022 [67] | Afghanistan | Primary school children | Cross-sectional study | < 15 years | 1426 | 881 | 545 | Saline wet mount method |
|  | Ahmed et al.2003 [68] | Pakistan | Primary school children | Cross-sectional study | 5-12 years |  | NA | NA | Direct microscopy |
|  | Ali et al.2020 [69] | Pakistan | Schools’ children | Cross-sectional study | 5-10 years | 3600 | NA | NA | Direct microscopy, sedimentation and floatation technique |
|  | Khan et al.2020 [70] | Pakistan | School children | Cross-sectional study | 5-15 years | 400 | 324 | 76 | Wet mount technique |
|  | | | | | | | | | |
| **WESTERN PACIFIC**  **REGION (WPR)** | Jeyapraksam et al.2019 [71] | Malaysia | National school children | Cross-sectional study | 6-13 years | 139 | 66 | 73 | Direct smear and formol ether concentration |
|  | Gier et al.2016 [72] | Vietnam | Schools of provinances | Cross-sectional study | 6-9 years | 510 | 243 | 267 | Kato-Katz smear and ELISA |
|  | Hung et al.2016 [73] | Vietnam | Primary school children | Cross-sectional study | NA | 1206 | NA | NA | Kato Katz technique |
|  | Belizario et al.2014 [74] | Philippines | Secondary schools | Cross-sectional study | 14-15 years | 633 | 243 | 390 | Kato Katz technique |
|  | Liu et al.2015 [75] | China | Elementary schools | Cross-sectional study | 9-11 years | 2179 | 1177 | 1002 | Kato Katz technique |
|  | Mekhlafi et al.2007 [76] | Malaysia | Primary school children | Cross-sectional study | 7-12 years | 292 | 145 | 147 | Kato Katz technique |
|  | Lee et al.2021 [77] | Solomon Islands | School children | Cross-sectional study | 6-14 years |  | NA | NA | Kato Katz technique |
|  | Ahmed et al.2011 [78] | Malaysia | Primary school children | Cross-sectional study | 6-13 years | 254 | 124 | 130 | Kato Katz technique |
|  | Belizario et al.2011 [79] | Philippines | Primary school children | Cross-sectional study | 5-10 years | 572 | 249 | 323 | Kato Katz technique |
