# Supplementary material for: Prevalence and correlates of soil-transmitted helminths in schoolchildren aged 5 to 18 years in low- and middle-income countries: a systematic review and meta-analysis
Source: Front Public Health. 2024 Mar 21;12:1283054. doi: 10.3389/fpubh.2024.1283054 (PMC10991833; doi:10.3389/fpubh.2024.1283054)

**< Supplementary table: 6> forest plot of prevalence of STH in different regions of included studies**

1. **Prevalence of STH in Africa region**


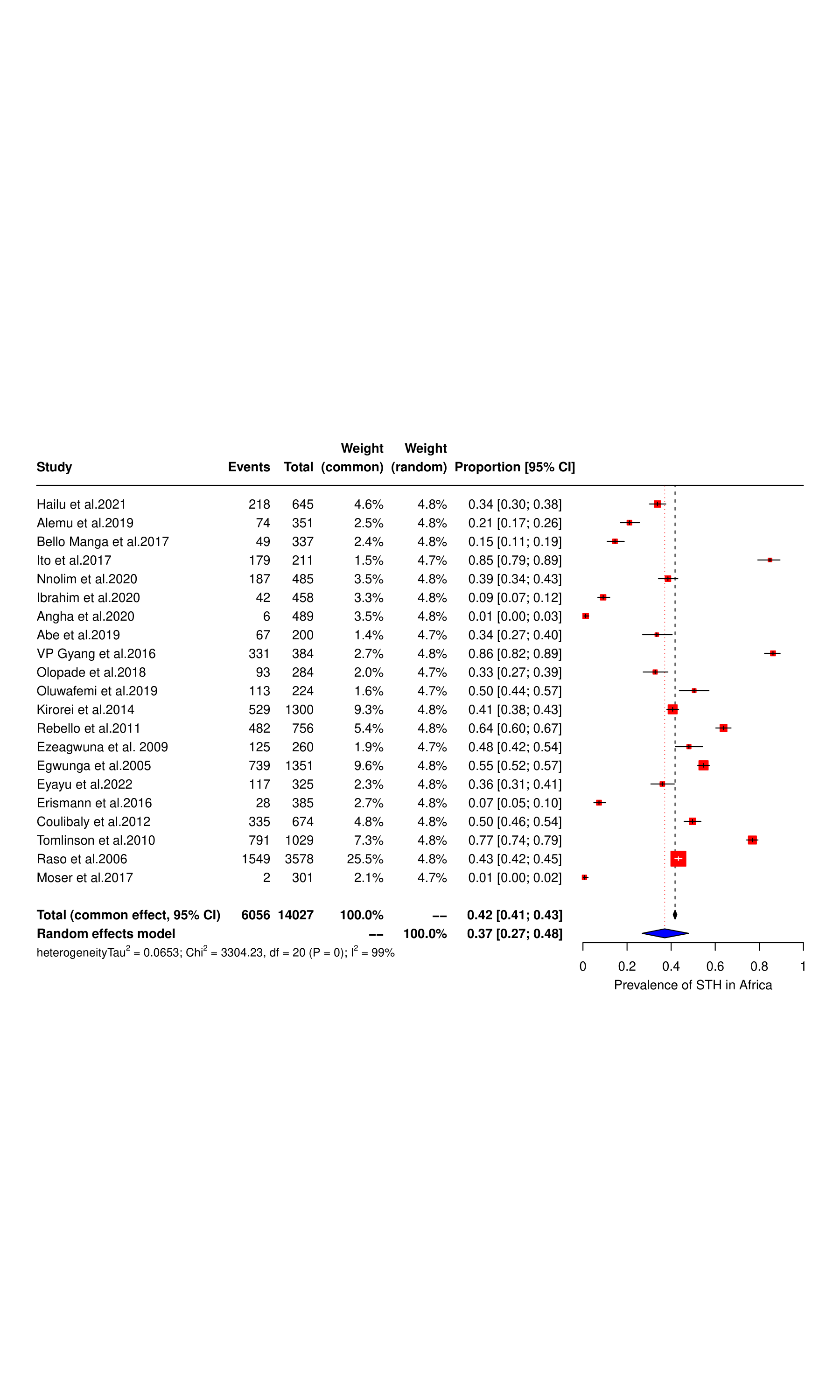


1. **Prevalence of STH in Eastern Mediterranean region**


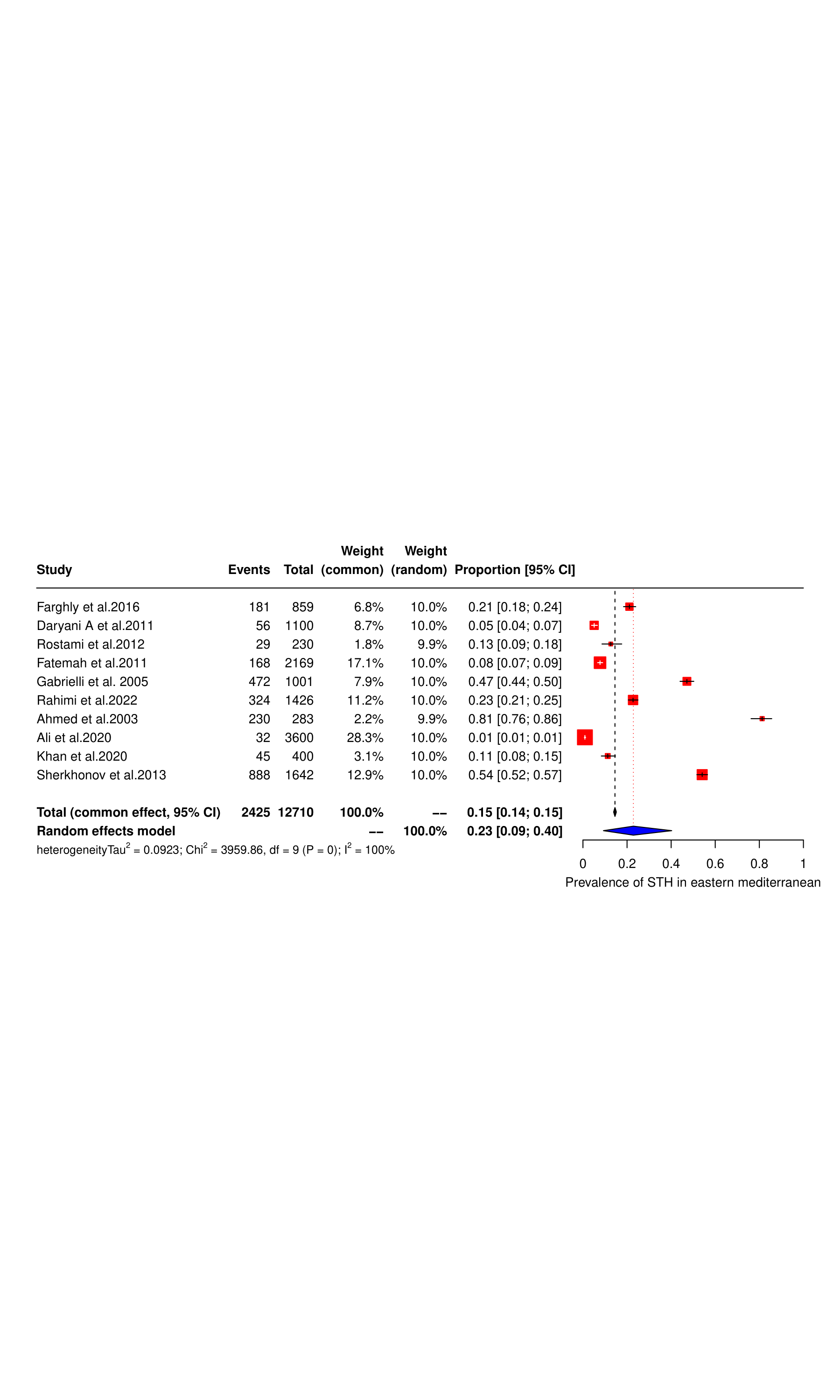


1. **Prevalence of STH in Europe region**


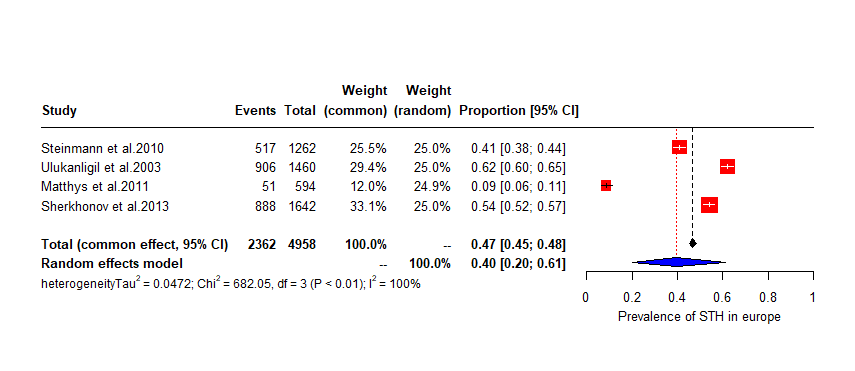


1. **Prevalence of STH in South East Asia region**


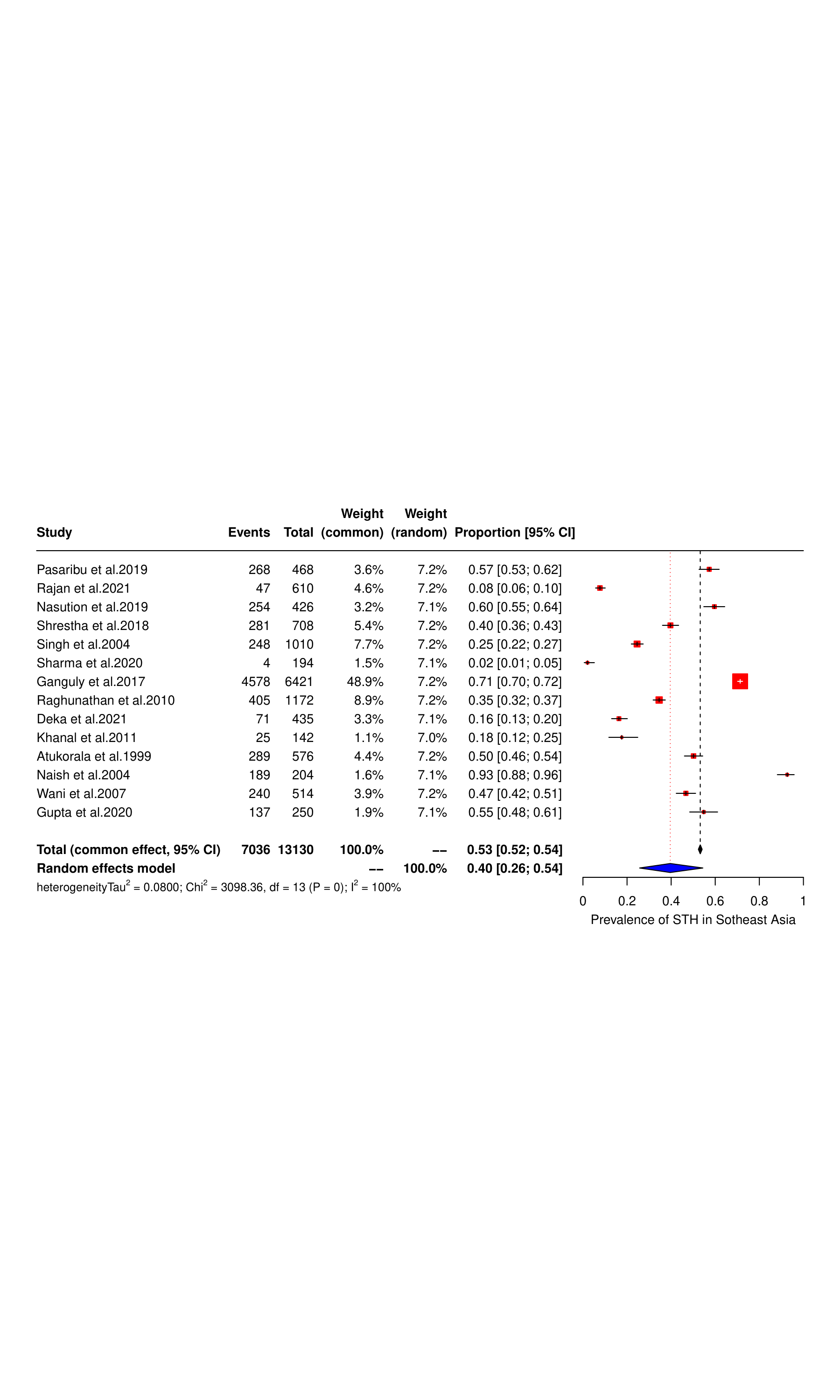


1. **Prevalence of STH in Western Pacific region**


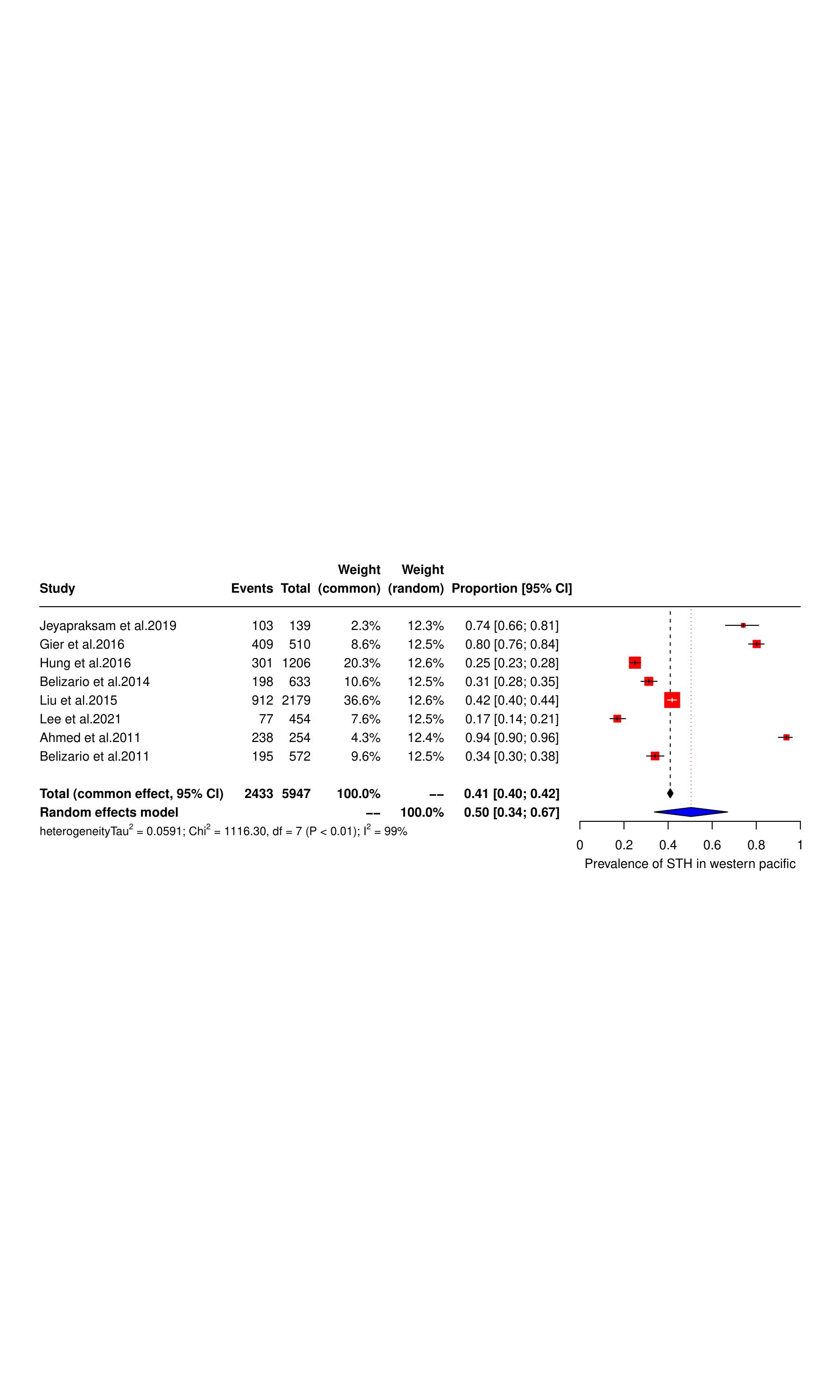

Supplement: Supplementary file 6 [file Table_6.docx]
