# Supplementary material for: Prevalence and correlates of soil-transmitted helminths in schoolchildren aged 5 to 18 years in low- and middle-income countries: a systematic review and meta-analysis
Source: Front Public Health. 2024 Mar 21;12:1283054. doi: 10.3389/fpubh.2024.1283054 (PMC10991833; doi:10.3389/fpubh.2024.1283054)
Supplement: Supplementary file 7 [file Table_7.docx]

**< Supplementary file:7> forest plot of prevalence of different STH species of included studies**

1. **Pooled prevalence of A. Lumbricoides among school going children in LMICs**

**
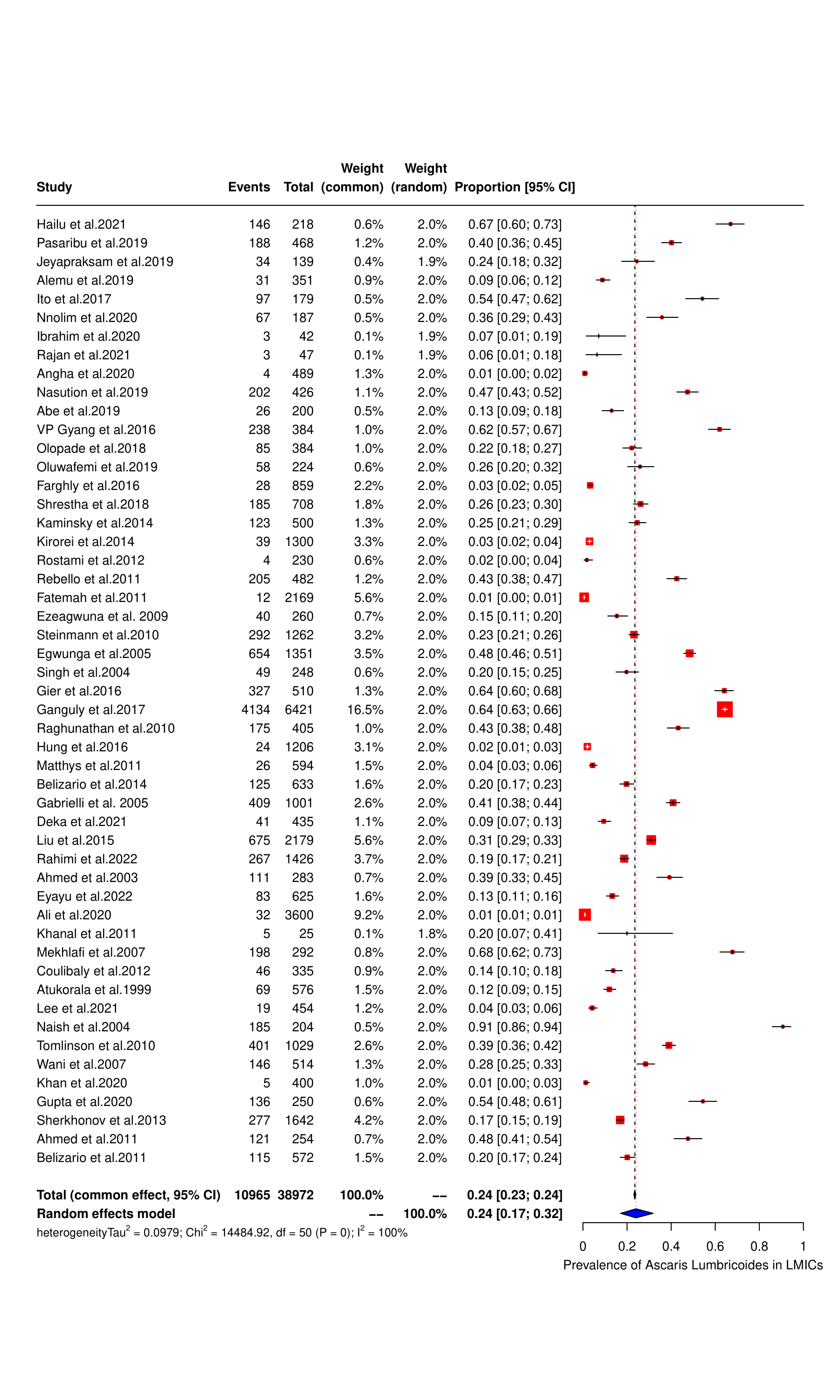
**

1. **Pooled prevalence of Hookworm among school going children in LMICs**

**
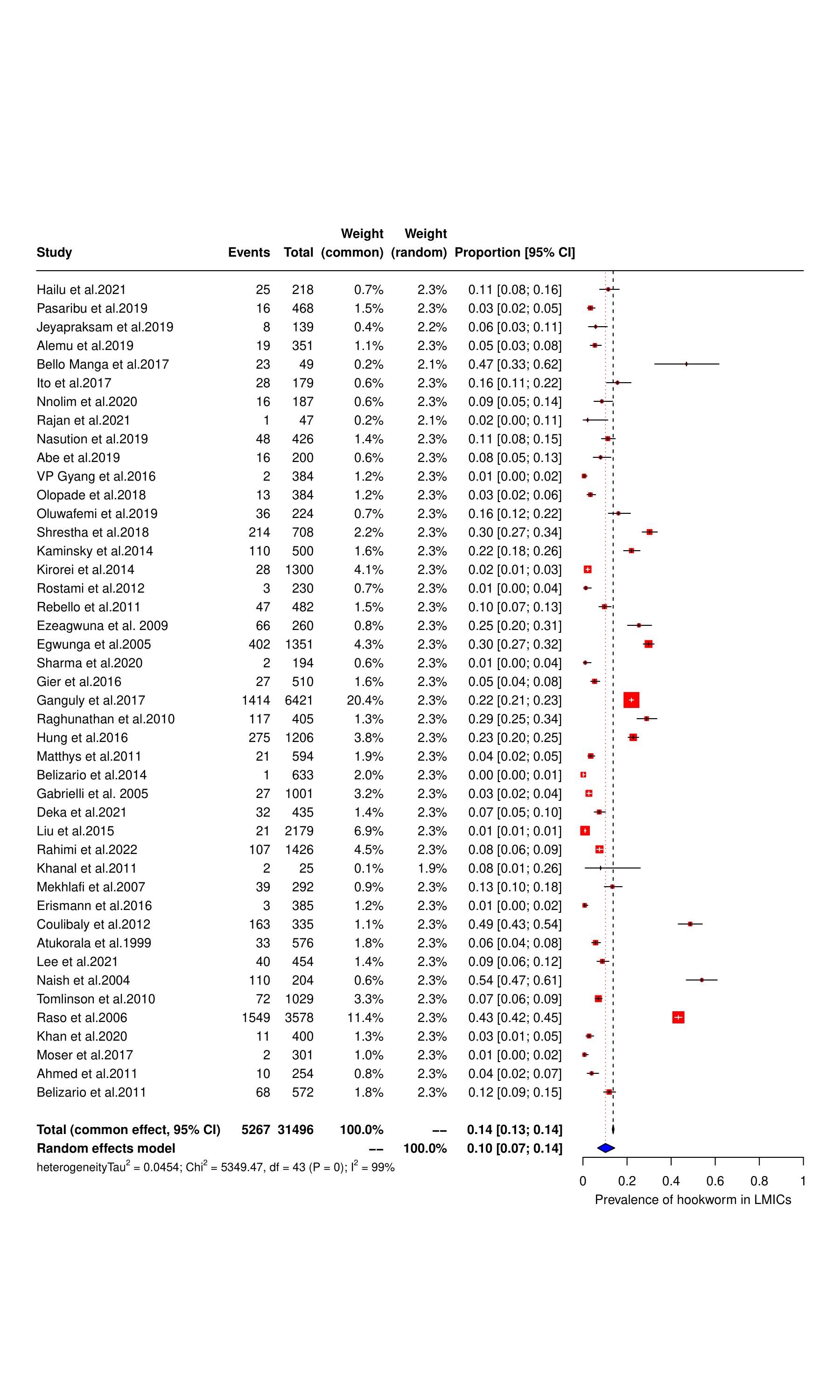
**

1. **Pooled prevalence of Trichiura Trichuris among school going children in LMICs**

**
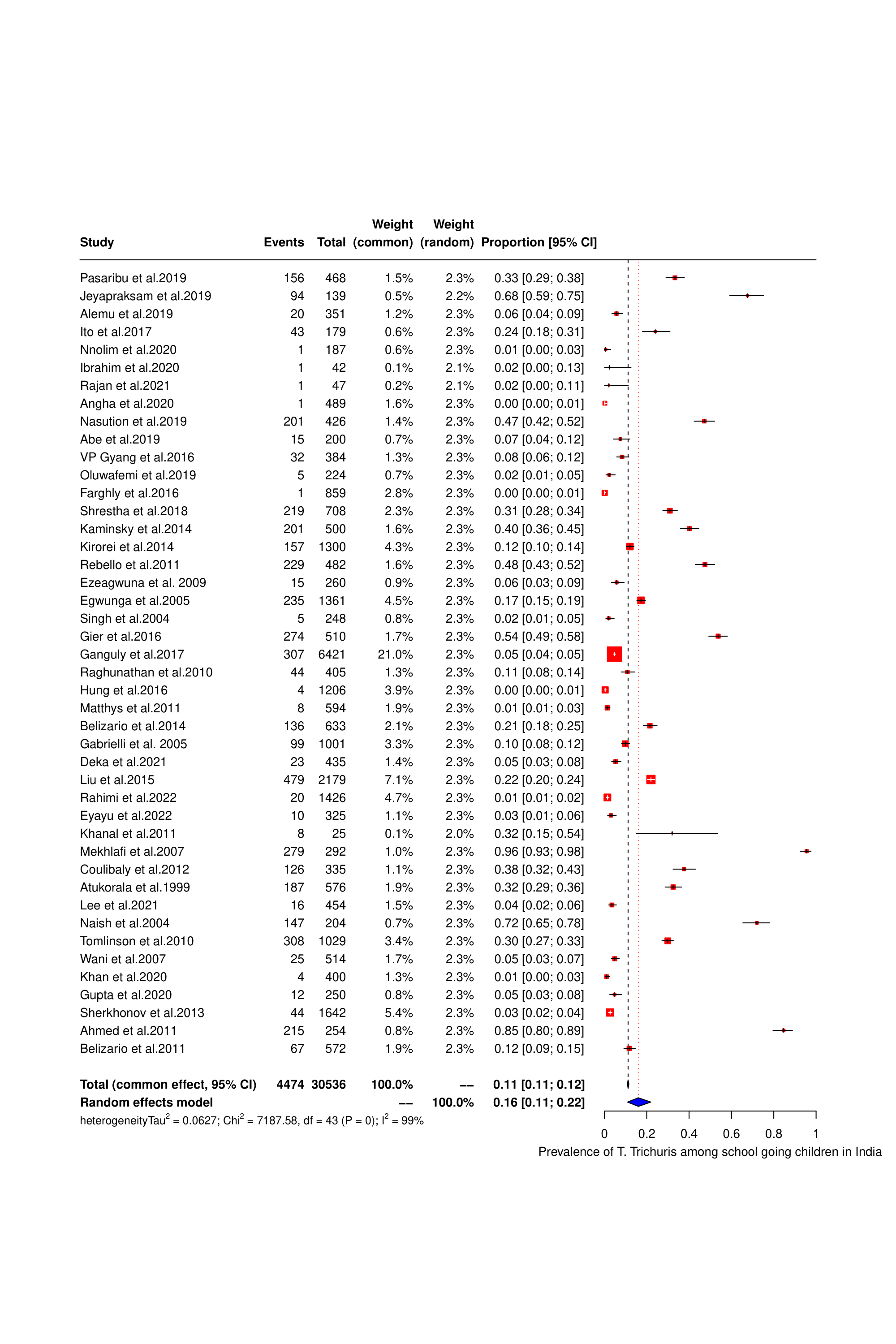
**
